# Single-Cell Transcriptomics Reveals Conserved Regulatory Networks in Human and Mouse Interneuron Development

**DOI:** 10.3390/ijms24098122

**Published:** 2023-05-01

**Authors:** Francesca Keefe, Jimena Monzón-Sandoval, Anne E. Rosser, Caleb Webber, Meng Li

**Affiliations:** 1 Neuroscience and Mental Health Research Institute, School of Medicine, Cardiff University, Cardiff CF24 4HQ, UK; 2 UK Dementia Research Institute Cardiff, Cardiff University, Cardiff CF24 4HQ, UK; 3 Division of Neuroscience, School of Bioscience, Cardiff University, Cardiff CF10 3AX, UK

**Keywords:** cortical interneuron, development, medial ganglionic eminence, human foetal brain, scRNA-seq, transcription regulatory network

## Abstract

Inhibitory GABAergic interneurons originate in the embryonic medial ganglionic eminence (MGE) and control network activity in the neocortex. Dysfunction of these cells is believed to lead to runaway excitation underlying seizure-based neurological disorders such as epilepsy, autism, and schizophrenia. Despite their importance in heath and disease, our knowledge about the development of this diverse neuronal population remains incomplete. Here we conducted single-cell RNA sequencing (scRNA-seq) of human foetal MGE from 10 to 15 weeks post conception. These MGE tissues are composed of largely cycling progenitors and immature post-mitotic interneurons with characteristic regional marker expression. Analysis of integrated human and mouse MGE data revealed species-conserved transcriptomic profiles and regulatory programs. Moreover, we identified novel candidate transcription regulators for human interneuron differentiation. These findings provide a framework for in vitro modelling of interneuron development and a strategy for potentially enhancing interneuron production from human pluripotent stem cells.

## 1. Introduction

The gamma-aminobutyric acid containing (GABAergic) inhibitory interneurons represent approximately 20% of the entire neuronal population in the human neocortex. Despite the small proportion, it is apparent that interneurons play a pivotal role in most, if not all, cortical functions [1,2,3]. Interneurons are required for maintaining the appropriate excitation–inhibition balance within the neocortex while the connectivity of a single interneuron onto multiple post-synaptic targets allows a synchronised, rhythmic pattern of firing [2,3,4]. The synchronised firing pattern generates network oscillations necessary for stable, long-distance transmission [5,6]. Unsurprisingly, loss of this homeostasis has been implicated in a number of neurological disorders, including autism spectrum disorders, schizophrenia, and epilepsies [2,3,4,5,7,8].

Interneurons are primarily born in two neighbouring structures of the developing ventral telencephalon, namely the medial and caudal ganglionic eminences (MGE and CGE), respectively [9,10,11]. They consist of three major subtypes based on their expression of somatostatin (SST), parvalbumin (PV), and ionotropic serotonin receptor 5HT3a (5HT3aR), respectively, and are highly heterogeneous in morphology and function [12,13]. Of these, the MGE-derived PV interneurons appear to be preferentially affected in neurodevelopmental disorders [1,14,15,16,17,18,19]. Thus, there has been growing interest in generating cortical interneurons from human pluripotent stem cells for disease modelling and drug screening [20,21,22,23,24,25]. These efforts are facilitated by recent advances in single-cell transcriptomics analysis of the developing and adult interneurons which have begun the identification of transcription factors contributing to cortical interneuron subtype fate determination in mice [26,27,28]. However, our understanding about the transcriptional program controlling interneuron differentiation remains limited.

Here we present a single-cell transcriptome analysis for human embryonic MGE of post conceptional weeks (PCWs) 10 to 15. Our finding provides support for the emergence of interneuron subtype diversification during human MGE development. Moreover, integrating mouse and human MGE transcriptional profiles identified a cross-species concordance in MGE development and identified potential novel regulators for interneuron differentiation.

## 2. Results

### 2.1. Developmental Dynamics of Human Fetal MGE

**Figure 1:**
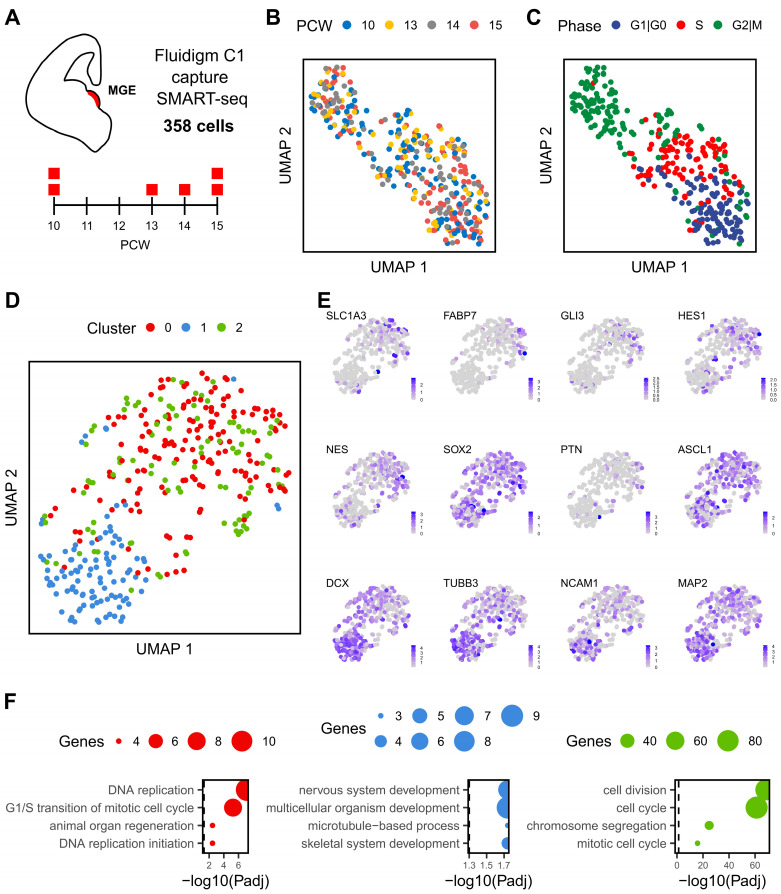
Transcriptional landscape of human MGE. (**A**), Single-cell RNA sequencing of human MGE, up to two samples were taken from 10 to 15 PCWs as illustrated in the timeline. A total of 358 cells passed the quality control and were analysed. (**B**), UMAP visualisation of cells coloured by post conceptional week. (**C**), UMAP visualisation of cells coloured by cell cycle phase. (**D**), UMAP after the difference between cell cycle scores (S-G2M) was regressed out from the data, colours indicate each of the three unbiased clusters. (**E**), Expression profile of neural progenitor and neuronal genes visualised by UMAP. (**F**), Top four biological processes enriched among the 64, 34 and 198 gene markers that characterise cluster 0, 1 and 2, respectively (from left to right). Gene Ontology (GO) terms were ordered based on their lowest adjusted *p* value. Dot size indicates the number of genes annotated to each GO term while dashed line indicates an adjusted *p* value threshold of 0.05.

We employed the Fluidigm C1 platform and SMART-Seq2 technology to study the transcriptomic profile of individual cells of human foetal MGE (Figure 1A). Of the 358 qualifying cells corresponding to six embryos of 10 to 15 PCWs, we obtained a mean of ~2 million counts per cell and detected a median of 6208 protein-coding genes per cell (Figure S1A–C). To visualise the dynamics of these cells, we performed principal component analysis using the top 1000 most variable genes (see Methods). Uniform Manifold Approximation and Projection (UMAP) for dimensionality reduction revealed a weak segregation amongst cells of different embryonic stages, suggesting MGE cells of 10–15 PCWs are transcriptionally similar with each other (Figure 1B). However, there was a clear segregation by cell cycle phases with subtle differences in the proportion of cells at each phase between the 10 and 15 PCW samples (Figure 1C and Figure S1D).

We then analysed the gene expression using an alternative workflow of Seurat to remove the effect of cell cycle-dependent genes on clustering and identified three main clusters between the four developmental stages of foetal MGE cells (Figure 1D). Expression of neural progenitor mark genes was enriched in cluster 0 and 2, while neuronal gene markers were preferentially detected in cluster 1 (Figure 1E). This differential gene expression pattern is consistent with the cell cycle status where cluster 0 and cluster 2 cells are mostly in S or G2/M phases while cluster 1 is constituted mainly by cells in G0/1 phases (Figure S1E). Between the two progenitor populations, cluster 0 was marked by genes involved in DNA replication (including Helicase, HELLS; Proliferating Cell Nuclear Antigen, PCNA; and Ran-specific GTPase-activating protein, RANBP1), while cell division and cell cycle-related genes, such as Karyopherin Subunit Alpha 2 (KPNA2) and Ubiquitin Conjugating Enzyme E2 C (UBE2C), characterised cluster 1 (Figure 1F and Figure S2). In contrast, genes involved in terminal neuronal differentiation and synaptogenesis such as Doublecortin (DCX), Stathmin 2 (STMN2), and Synaptotagmin 1 (SYT1) were enriched in cluster 1 cells, consistent with cell differentiation and nervous system development being the most significantly enriched biological process of this cluster (Figure 1F and Figure S2).

### 2.2. Transcriptional Heterogeneity and Emergence of Interneuron Subtypes

**Figure 2:**
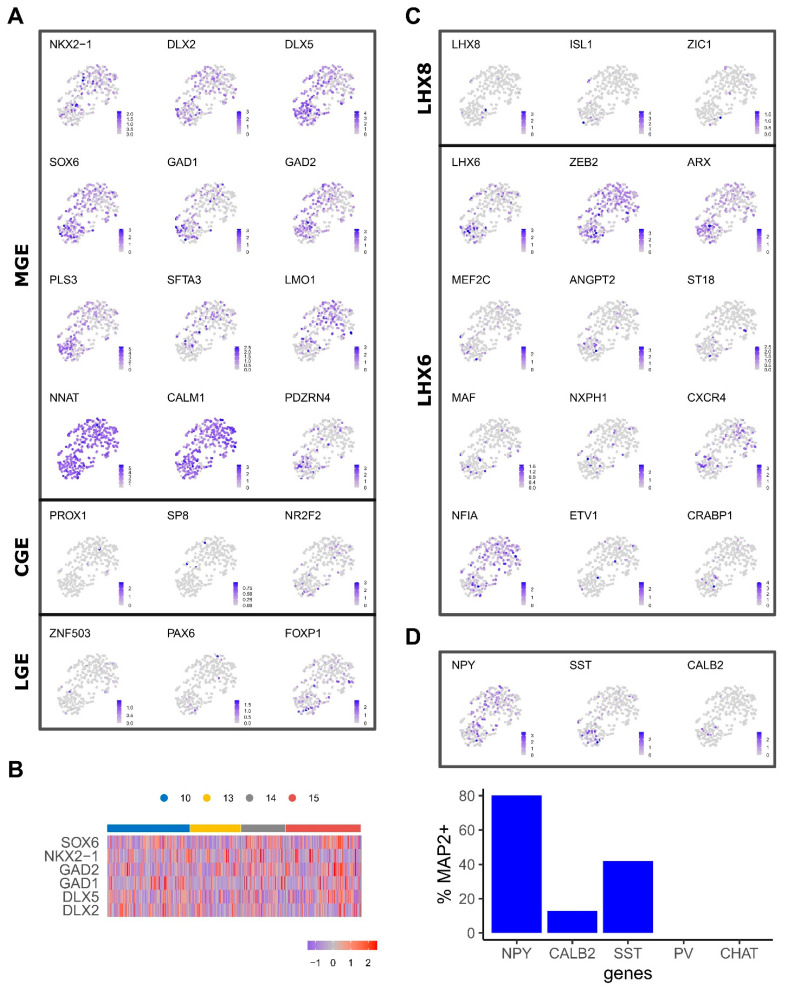
Expression of regional and interneuron subtype genes. (**A**), UMAP coloured by the gene expression levels of MGE, LGE and CGE markers. (**B**), Heatmap shows gene expression patterns of selected genes across PCW time points. (**C**), UMAP coloured by the gene expression of markers that characterise interneuron LHX6 and LXH8 populations. (**D**), Gene expression patterns of post-mitotic interneuron markers NPY, SST, CALB2. Bar plot indicates the percentage of neurons that express each marker (relative to the MAP2-expressing cells).

Transcription factor NKX2.1 is specifically expressed in MGE progenitors and post-mitotic MGE-derived interneurons that reside in the striatum. *NKX2.1*^+^ cells were highly enriched in samples of all ages and were more abundant in clusters 0 and 2 (Figure 2A). Consistent with the MGE identity of our cells, transcripts that are highly specific to LGE and CGE or cortex, such as *ZNF503* and *PROX1*, respectively, were rarely detected (Figure 2A). Moreover, expression of other MGE and interneuron developmental regulators correlated well with differentiation and cell cycle status. For example, *FOXG1^+^*, *DLX2*^+^ and *DLX5*^+^ cells were distributed across 10–15 PCW MGE samples while *SOX6*, *GAD1* and *GAD2* were preferentially expressed in the neuronal cluster (Figure 2A).

In mice, Nkx2.1^+^ progenitors give rise to cholinergic interneurons (ChAT^+^) under the control of Lhx8 and GABAergic interneurons marked by Lhx6, which later differentiate into parvalbumin (PVALB) and somatostatin (SST) subtypes. *maf* and *mef2c* have been suggested to have preferential regulatory roles for SST and PV fates, respectively [27,28]. *MEF2C* and *MAF* were detected in a proportion of *LHX6^+^* cells mostly in the neuronal cluster where *SST^+^* and neuropeptide Y (*NPY^+^*) cells were also found (Figure 2B). On the other hand, cells expressing *LHX8* and other cholinergic regulators (eg. *ISL1*, *ZIC1*) were detected albeit in fewer number of cells compared to *LHX6* (Figure 2B) [29,30]. However, *ChAT*^+^ neurons were not detected. These observations either reflect a later onset of cholinergic development or the relative representation of the LHX6 and LHX8 interneuron lineages.

Repression of Nkx2.1 by Zeb2 in mouse MGE progenitors confers cortical interneuron fate, while in its absence these progenitors progress into striatal interneurons retaining Nkx2.1 expression [31]. *ZEB2* exhibits a heterogeneous expression pattern across the clusters while a mosaic expression of striatal interneuron indicator genes such as *ETV1* and *NFIA* was also observed. Together, these findings indicate the emergence of cortical interneuron subtypes and spatial specification.

The single-cell gene expression presented above is supported by immunostaining of class-defining markers in dissociated 15 PCW MGE tissue (Figure S3). After 2 days in culture, a significant proportion of the population was found to express progenitor markers FOXG1 and NKX2.1 (42 ± 15% and 25 ± 4.1%, respectively). Cells marked by post-mitotic interneuron subtype markers, such as SST (0.4% ± 0.1), ChAT (1.1 ± 0.08%) and Calretinin (encoded by CALB2, 18 ± 3%), were also readily detected along with cells stained positive for pan-neuronal marker MAP2 and NeuN (25 ± 4.5%, 17 ± 3.3%, respectively). Consistent with scRNA-seq data, no PVALB-positive cells were identified.

### 2.3. Conserved Biological Processes between the Human and Mouse MGE

**Figure 3:**
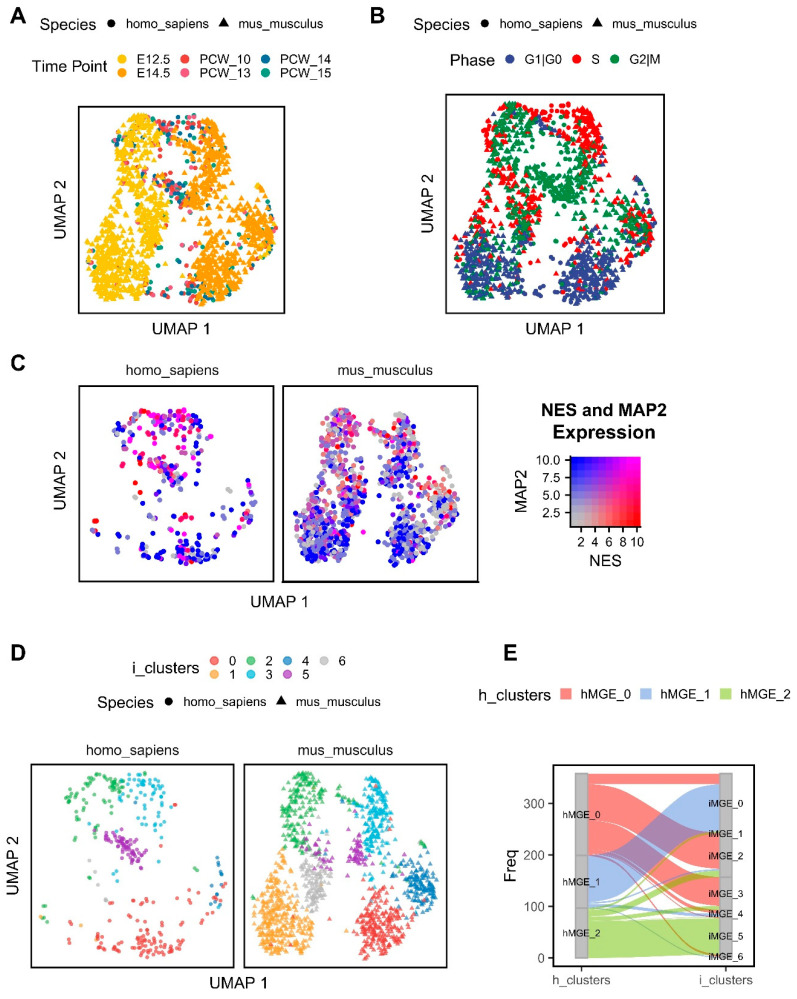
Integration of human and mouse MGE gene expression data and identification of conserved cell clusters. UMAP was calculated based on the first 30 principal components of the top 3000 most variable genes. (**A**), UMAP of the integrated human and mouse data has been coloured by time points on embryonic days E12.5 and E14.5 (mouse) and 10–15 PCWs (human), different shapes denote each species. (**B**), UMAP coloured by cell cycle phase with shapes identifying each species. (**C**), UMAP coloured by the gene expression levels of NES and MAP2 on the SCT normalised dataset, plot has been divided into a single panel per species. (**D**), UMAP coloured by the integrated cell clusters from the MGE (mouse and human, denoted as i-clusters), again each species has been plotted in a separate panel. (**E**), Alluvial plot showing the correspondence between the cell clusters identified only in the human dataset (h-clusters) and their correspondence to the integrated clusters based on the combined analysis of human and mouse data.

We next integrated the human MGE scRNAseq data with that previously published from the mouse MGE based on the shared one-to-one ortholog (see Methods) [28]. The integrated dataset consisted of 1614 cells and 10,362 genes from the MGE across two species. The mouse MGE cells from embryonic day 12.5 (E12.5) and E14.5 presented the largest variance and clustered separately. The 10–15 PCW human MGE cells were, however, more evenly distributed (Figure 3A). A strong segregation of cycling and non-cycling cells based on their cell cycle phase is consistent between the two species (Figure 3B) and is in agreement with differential expression of NES and MAP2 (Figure 3C).

Seven cell clusters were identified in the integrated human and mouse MGE dataset with a variable number of cells from each species (Figure 3D). We observed a good correspondence between the clusters previously identified in the human MGE alone (Figure 1D) and those in the integrated dataset (Figure 3E). For example, human cluster 1 mostly corresponds to the integrated cluster 0 (i-cluster)/post-mitotic neurons. Using the *FindMarkers* function of *Seurat*, we identified conserved genes between the human and mouse in a given cell cluster (Table S1), all of which were detected in at least 25% of cells. Gene ontology (GO) enrichment analysis on the conserved genes of i-cluster 0 highlighted genes involved in dendrite morphogenesis, regulation of neuron projection development, axonogenesis, cell adhesion and neuron migration (Figure S4A). In turn, integrated clusters 2 and 3 are marked by the enrichment of genes involved in DNA replication (Figure S4B,C). Mitochondrial/metabolic-associated genes were heavily enriched in cluster 4 (Figure S4D). Finally, species integrated cluster 5 contained cells highly expressing mitotic cell cycle-associated genes (Figure S4E). There were no significantly enriched biological processes among the 42 and 41 conserved genes of the integrated clusters 1 and 6 when using the applied threshold.

### 2.4. Gene Regulatory Network Inference with SCENIC Reveals Novel Human Candidate Interneuron Regulators

**Figure 4:**
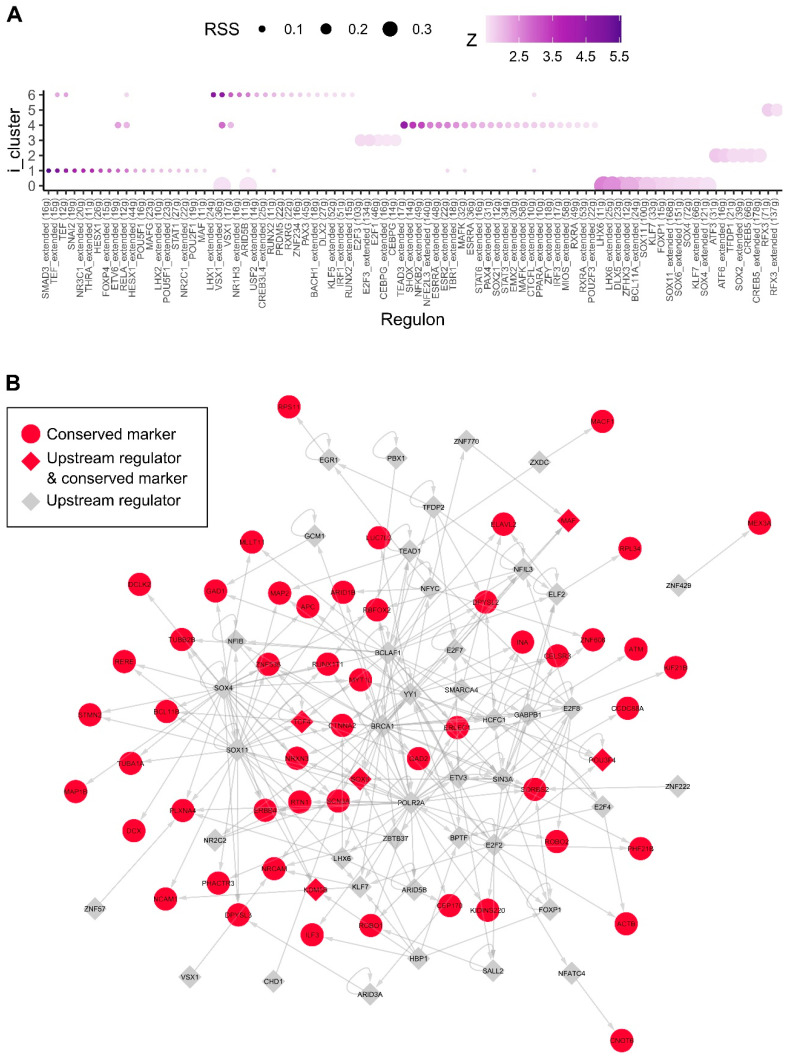
Human active regulons within the integrated MGE clusters. We inferred the gene regulatory network based on co-expression and TF binding site enrichment separately for human MGE using SCENIC. (**A**), The regulon specific scores (RSS) allow us to identify active regulons in the integrated MGE cell clusters. (**B**), Inferred regulatory network showing the high confidence links among the conserved markers in the integrated neuronal cluster 0 and their immediate upstream regulators. Genes are coloured in red if they were identified as conserved gene markers in the integrated mouse/human MGE cluster 0. Transcription factors are shaped in diamonds.

We then employed the SCENIC (Single-Cell rEgulatory Network Inference) tool to construct gene regulatory networks (or regulons) consisting of a set of transcription factors and their target genes that are most likely active within the cell clusters defined in the integrated mouse and human MGE [32]. This revealed a network of SMAD3 containing regulons in progenitor cluster 1 (Figure 4A). Interestingly, TGFβ signalling has been shown previously to be required for regulating NKX2.1^+^ progenitor cell cycle exit and their differentiation into LHX6^+^ interneurons [25], hence providing direct experimental validation for this prediction.

Moreover, SCENIC identified a neuronal dominant cluster (i-cluster 0) containing regulons that correspond to 11 transcription factors and their target genes (Figure 4A). These include known interneuron specification genes, such as the SRY-Box Transcription Factor 6 (SOX6), LIM Homeobox 6 (LHX6), Distal-Less Homeobox 5 (DLX5) and Visual System Homeobox 1 (VSX1), as well as transcription factors known to be important in neurogenesis, such as SRY-Box Transcription Factor 4/11 (SOX4 and SOX11). In addition, we identified transcription factors B-Cell Lymphoma/Leukaemia 11A (BCL11A/CTIP1), AT-Rich Interaction Domain 5B (ARID5B), Zinc Finger Homeobox 3 (ZFHX3) and Kruppel-Like Factor 7 (KLF7) as novel candidate regulators for interneuron differentiation and/or specification. The inferred gene regulatory network containing only high confidence links for the conserved gene markers of cluster 0 and their immediate upstream regulators is shown in Figure 4B. When compared to the inferred regulatory network based on mouse data only, we also identified Sox6, Lhx6 and Klf7 as active regulators on the cells corresponding to the neuronal integrated cluster 0, indicating conserved regulatory elements in interneuron specification (Figure S5).

## 3. Discussion

An in-depth understanding of human cortical and striatal interneuron development is fundamental to decipher their function in the normal brain and how aberrant development and dysfunction of these cells may lead to neurodevelopmental disorders. This single-cell RNAseq study illustrates the heterogeneity and dynamic transcriptomic landscape of human foetal MGE cells and provides evidence on the conserved developmental trajectory of human and mouse MGE interneurons. Moreover, our study identified a novel set of transcription factors as potential regulatory networks governing human MGE interneuron differentiation.

With several competing theories in the literature, details concerning interneuron diversification into specific subtypes remain to be conclusively determined [33,34]. With the caveat of moderate cell numbers that limit prospective subtype clustering, cells expressing prospective MGE-derived interneuron subtype regulators or predictive genes were detected in our MGE samples of all ages. Moreover, some subtype-committed cell populations (e.g., *NPY* and *SST*) were detected. Thus, data described here support the notion that, as in mice, lineage-dictated subpopulations emerge within the MGE [27,28,35,36]. However, we did not find *PVALB*^+^ and *ChAT*^+^ neurons in human MGE of 10–15 PCWs. This may be a reflection of the developmental period studied or due to them existing in very low numbers that we were not able to detect in our current analysis [34,37]. Alternatively, these subtypes may emerge primarily within the region of destination under the influence of local extrinsic cues [34,38].

Using the SCENIC tool on the integrated human and mouse MGE single-cell profiles, we identified several sets of transcription factors characteristic to defined i-Clusters. Albeit with a moderate regulon specificity score (RSS), implicating low cell type specificity, a *SMAD3*-containing regulatory network was predicted for neural progenitor i-Cluster 1. This cluster of cells is enriched with a DLX2-expressing progenitor population with another *SMAD* (*SMAD1*) as one of its signature genes (Table S1). Interestingly, TGFβ signalling, which acts through SMAD proteins, has been demonstrated as a novel regulatory pathway in modulating the maintenance of human ESC-derived NKX2.1^+^ progenitors and their differentiation into LHX6^+^ interneurons [25]. This prior study thus validates the SCENIC prediction. On the other hand, the transcription factors identified in this set provide a foundation for further investigation into detailed molecular mechanisms downstream of TGFβ control of interneuron progenitor expansion and cell cycle exit.

Moreover, the regulatory network inference identified a set of species-conserved developmental regulators such as LHX6, SOX6 and DLX5 being active in the neuronal dominant i-Cluster 9 (human cluster 1). These transcription factors have previously been applied successfully to generate interneurons from human pluripotent stem cells via direct programming [39]. Significantly, the regulons contain transcription factors not previously implicated in interneuron development, such as KLF7, BCL11A and ZFHX3. KLF7 has been reported to be capable of promoting axon outgrowth and olfactory sensory neurogenesis while BCL11A is required for spinal cord sensory circuit formation [40,41,42]. ZFHX3 encodes a transcription factor that has been shown to be capable of promoting neurite outgrowth [43]. Interestingly, *de novo* loss-of-function mutation of this gene has been suggested to cause intellectual disability [44]. The identification of ZFHX3 as a candidate interneuron regulator thus provides further support that abnormal interneuron development contributes to the pathogenesis of neural developmental disorders. Moreover, our findings present an opportunity to generate a more authentic subtype enriched with in vitro interneuron differentiation culture through the engineered expression of one or more of these transcription factors.

## 4. Materials and Methods

### 4.1. Tissue Preparation

Human foetal tissue was collected from medical terminations of pregnancy with full donor consent, through the SWIFT human foetal tissue bank (http://www.biobankswales.org.uk/swift-research-tissue-bank), under UK Human Tissue Authority research license (No. 12457) held by Cardiff University and with ethical approval of the project from the Bro Taf local research ethics committee. Gestational age was estimated through ultrasound scan prior to the procedure in combination with measurement of foetal regions [45]. MGE tissue was harvested from seven foetuses corresponding to approximately 10 and 12–15 post conception weeks (PCWs). MGEs were individually incubated at 37 °C for 20 min in TrypLE Express containing Donase-α. Tissue was dissociated by trituration in 200 μL of DMEM/D to obtain a quasi-single-cell suspension. The cell concentration was calculated by manual counting and viability was measured by Trypan Blue assay.

### 4.2. Single-Cell Capture, Library Preparation and Sequencing

Single cells were captured according to the manufacturer’s protocol on a C1 Single-Cell IFC microfluidic chip designed for 10 to 17 μm cells (Fluidigm, Inc., South San Francisco, CA, USA). This captured one cell in each of up to 96 capture chambers. Capture efficiency and cell viability were determined by microscopy before cDNA amplification and library preparation using the SMART-Seq 46 v4 Ultra Low Input RNA kit chemistry (Clontech, Mountain View, CA, USA) and Nextera XT DNA Library Prep Index Kit (Illumina, San Diego, CA, USA). Single-cell sequencing was carried out on the HiSeq 4000 Illumina platform with the following parameters: paired-end reads, read length 2 × 75 bp and read depth of approximately 2 million reads per cell.

### 4.3. Data Processing

The quality of the sequences was assessed using FastQC (version 0.11.8) and results were summarised using MultiQC. After removing adapters by TrimGalore (version 0.5.0), transcript abundances were quantified using Kallisto (version 0.46.0) [46]. The reference index used include all cDNA and ncRNA sequences from Ensembl (release 99) as well as the Array Control RNA spike sequences from Thermo Fisher. Sequences in scaffold chromosomes were removed and we kept only chromosomes 1–22, X and Y. We removed cells with less than 25% of reads mapping to protein-coding genes and retained cells with at least half a million reads. We further removed an outlier sample from 12 PCWs. We filtered out lowly expressed genes, keeping only those with more than 5 counts in at least 1% of the cells. The filtered dataset consisted of 14,673 protein-coding genes across 358 cells.

### 4.4. Dimensionality Reduction

Gene expression data were logNormalized using Seurat function and a scale factor equal to 10,000. Principal component analysis was based on the top 1000 most variable features (vst method). We identify cell clusters based on the first 10 principal components using the *FindCluster* function with a resolution equal to one in first instance and equal to 0.5 after regressing out the difference in cell cycle scores. Similarly, Uniform Manifold Approximation and Projection (UMAP) was based on the first 10 principal components. We followed the alternative workflow to keep differences between cycling and non-cycling cells by regressing out the differences between the S and the G2M scores, based on the updated (2019) cell cycle gene annotations from Seurat.

### 4.5. Differential Expression Analysis

We used the *FindMarkers* function from Seurat to obtain gene markers for each of the four human MGE clusters, including only positive markers detected in at least 25% of cells and with a minimum logFC of 0.25. For further analysis, we only considered gene markers with an adjusted *p* value < 0.05.

### 4.6. Gene Ontology Enrichment Analysis

GO annotations were retrieved from Ensembl through *biomaRt*, and we segregated annotations according to three main branching categories: biological processes, cellular component and molecular function. We only focused on GO terms with at least 20 genes in our gene background population (*n* = 14,673), and to assess GO enrichment we used a hypergeometric test. We adjusted for multiple testing and only GO terms with a false discovery rate < 0.05 were considered enriched.

### 4.7. Integration with Mouse MGE Dataset

Gene expression data from the mouse MGE were obtained from the GEO record (GSE109796), and cell annotations were kindly provided by Zhen Li and further analysis was based only on the MGE cells from their reported filtered dataset [28]. In order to integrate human-on-mouse gene expression data from the MGE, we focused only on *one-to-one* gene orthologs that were present in both datasets. This led us to a combined dataset of 1256 mouse and 358 human MGE cells across 10,362 genes. We used *SCTransform* from Seurat to integrate both datasets, based on the top 3000 most variable features and the first 30 principal components [47]. For conserved gene markers in the integrated dataset we used the *FindConservedMarkers* function form Seurat with species as the grouping variable, focusing only in positive markers, a min percentage of 25% cells and a logFC threshold of 0.25. We considered only conserved markers, those with a maximum combined *p*-value < 0.05.

### 4.8. Gene Regulatory Network Inference

To infer the gene regulatory network, we ran SCENIC [32] in R separately for the human and mouse MGEs [32]. Briefly, we used the GENIE3 algorithm to infer the gene regulatory network from the human MGE expression data, and regulons for a particular transcription factor were based on DNA motif analysis using *RcisTarget*. We calculated the regulon specificity scores (RSS) [48] to identify specific regulators within each of the species-conserved cell clusters based on the individual *AUCell* scores.

## Data Availability

The data discussed in this publication are deposited in NCBI’s Gene Expression Omnibus and are accessible through GEO Series accession number GSE230467 (https://www.ncbi.nlm.nih.gov/geo/query/acc.cgi?acc=GSE230467).

## Supplementary Materials

The following supporting information can be downloaded at https://www.mdpi.com/article/10.3390/ijms24098122/s1.
