# Supplementary material for: Single-Cell Transcriptomics Reveals Conserved Regulatory Networks in Human and Mouse Interneuron Development
Source: Int J Mol Sci. 2023 May 1;24(9):8122. doi: 10.3390/ijms24098122 (PMC10179417; doi:10.3390/ijms24098122)
Supplement: Supplementary file 1 [file ijms-24-08122-s001.zip › Figures S1-S5.pdf]

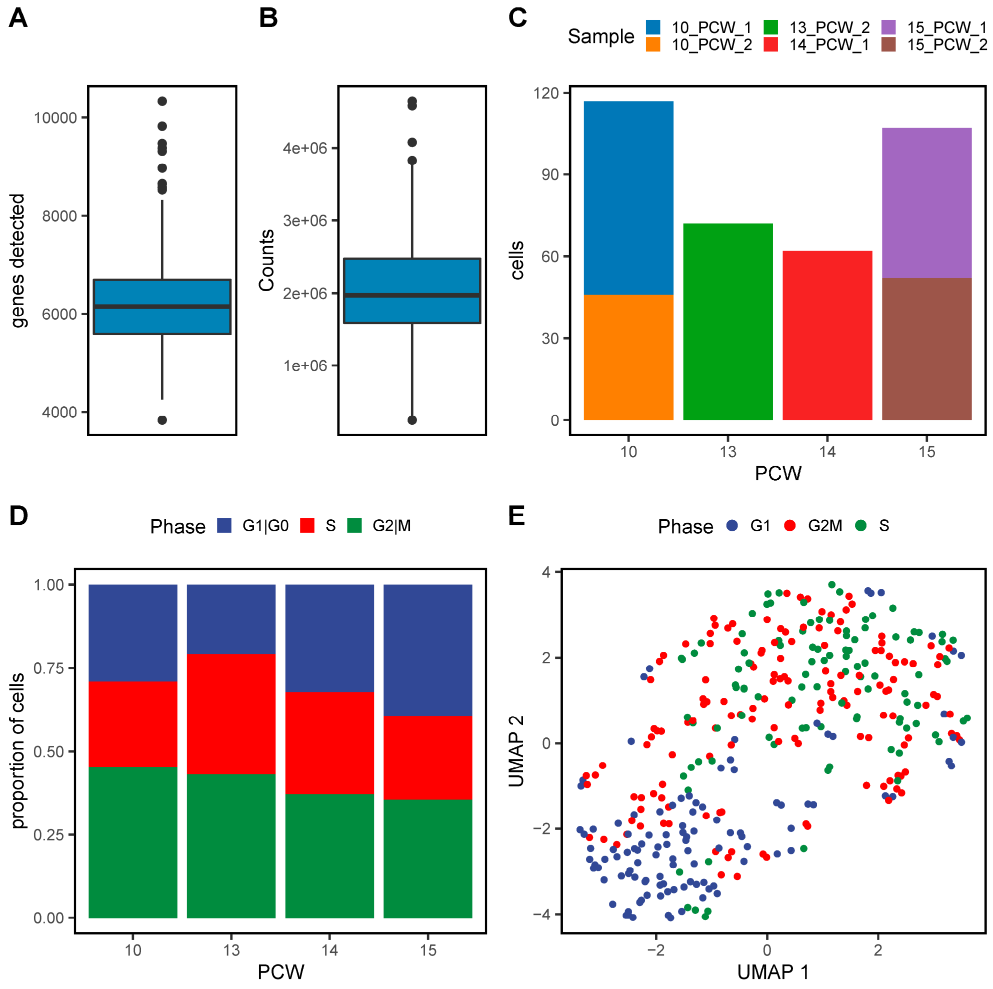

Figure S1. Overview of the filtered human MGE dataset and the effect of regressing out the differences in cell cycle score. A, Boxplot shows the number of protein-coding genes detected per cell. B, Boxplot shows the number of counts mapping to protein-coding genes. C, Number of cells from each individual MGE tissue sample at each PCW. D, Barplot shows the proportion of cells from each cell cycle stage in each PCW. We observed a different proportion than expected by chance at PCWs 10 and 15 (Chi-squared test, PCW 10: X = 14.10308, $p = 0.0008660757$; PCW 13: X = 5.196778, $p = 0.07439333$; PCW 14: X = 2.538101, $p = 0.2810984$, PCW 15: X = 11.2093, $p = 0.003680707$). E, UMAP after regressing out the difference between cell cycle scores (S-G2M) has been coloured by cell cycle phase.

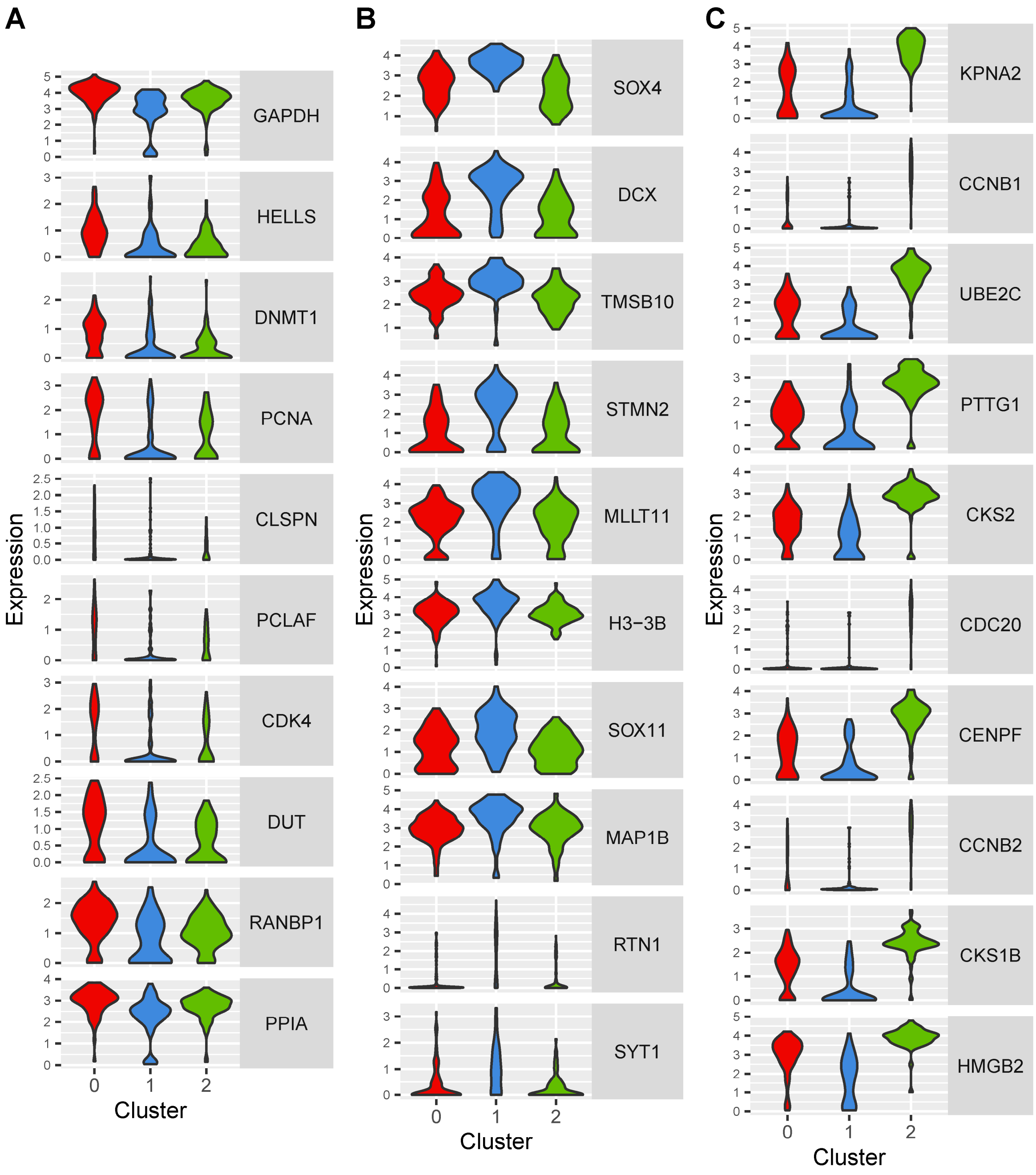

Figure S2. Gene markers characterising each of the human MGE clusters. Violin plots show the gene expression levels of the top 10 markers that characterised each unbiased cluster of the human MGE. A, Top 10 gene markers of cluster 0. B, Top 10 markers of cluster 1. C, Top 10 markers of cluster 2.

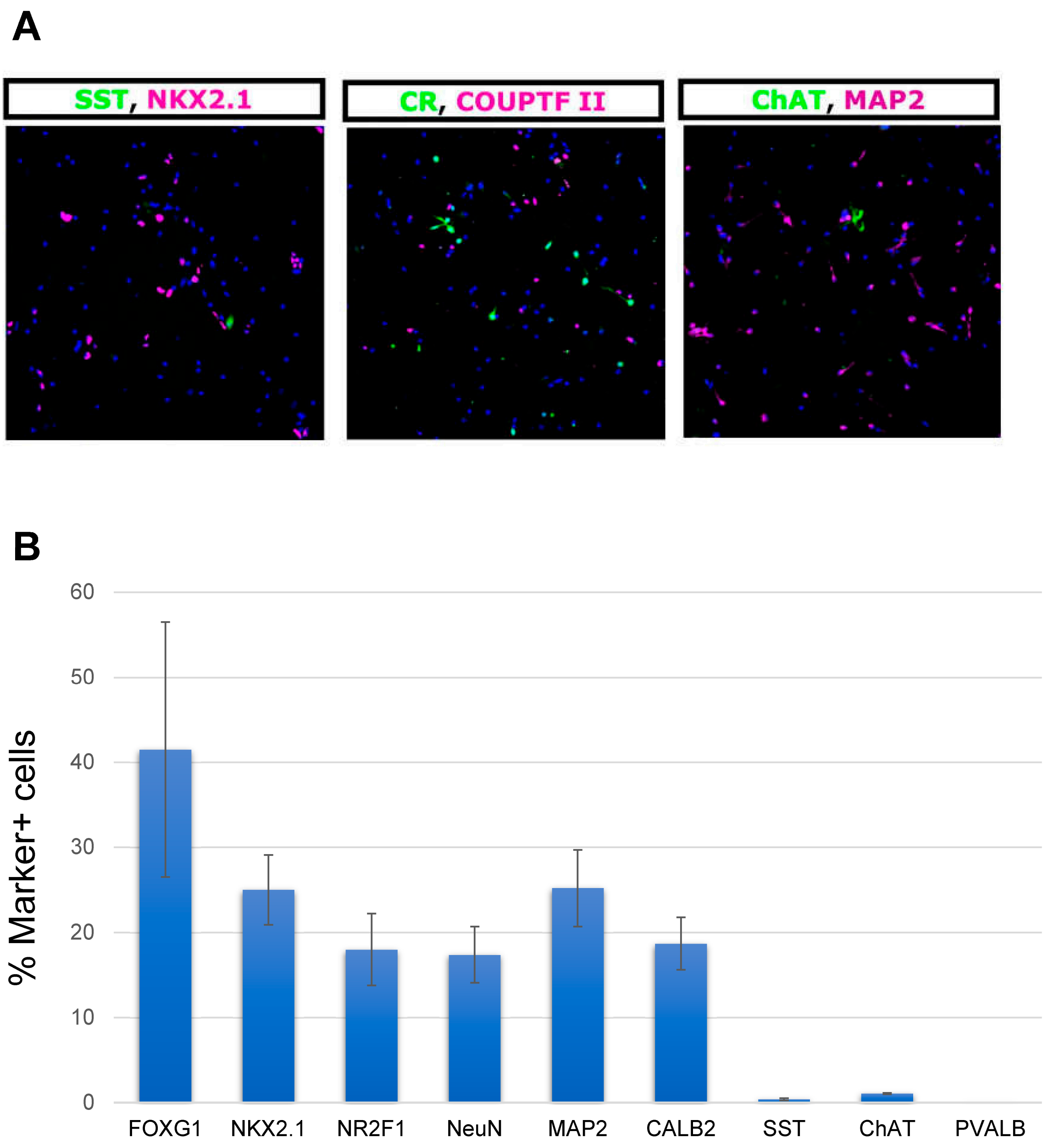

Figure S3. Immunocytochemical characterisation of primary human MGE cultures. Dissociated human MGE tissue of 15 PCWs was cultured for 2 days in vitro before fixing for immunostaining. A, Panel of immunohistochemistry deconvoluted images. B, Barplot shows the mean percentage of marker-expressing cells out of total cell population (DAPI+), along with +/− S.E.M. Data obtained from three replicate MGE cultures from one embryo.

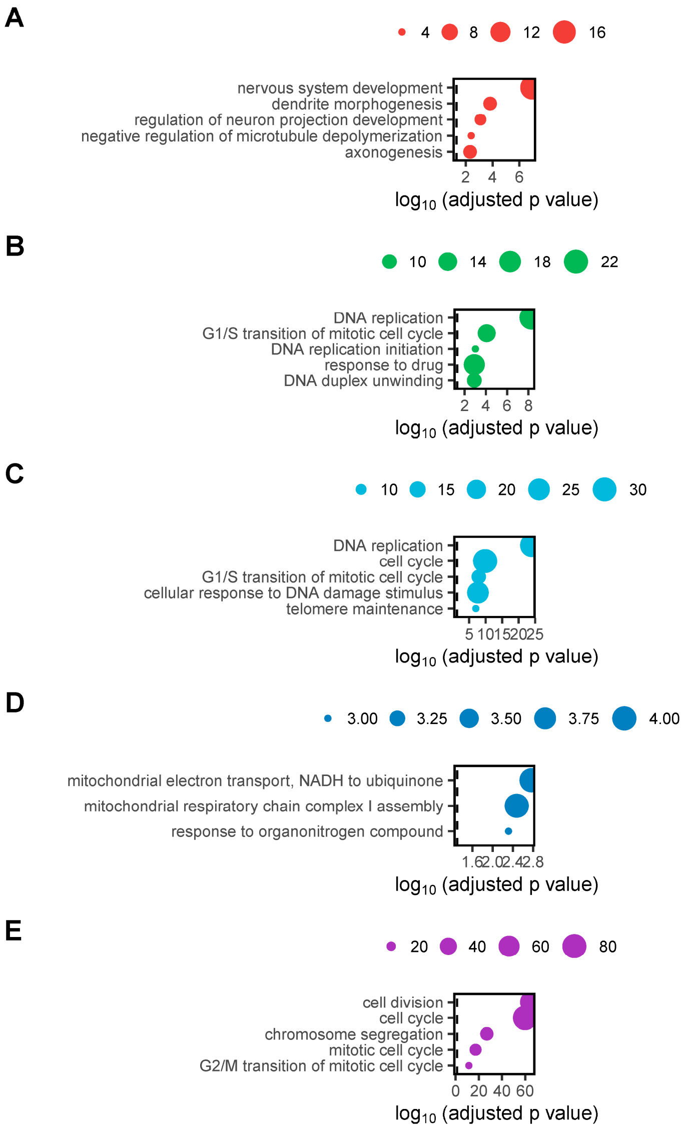

Figure S4. Top 5 enriched biological processes among conserved gene markers from the integrated human and mouse data. We identified conserved markers for each of the cell clusters in the species-integrated dataset. The dot plot shows the top 5 enriched biological processes among the conserved makers of A Cluster 0 (n = 76), B Cluster 2 (n = 259), C Cluster 3 (n = 130), D Cluster 4 (n = 24) and E Cluster 5 (n = 157). Gene Ontology terms are sorted by their associated adjusted p value. Dashed line indicates an adjusted p value of 0.05, only those GO terms with an adjusted p value < 0.05 are shown. Note that there were no enriched biological processes among the conserved markers of Cluster 1 (n = 42) and Cluster 6 (n = 41).

A

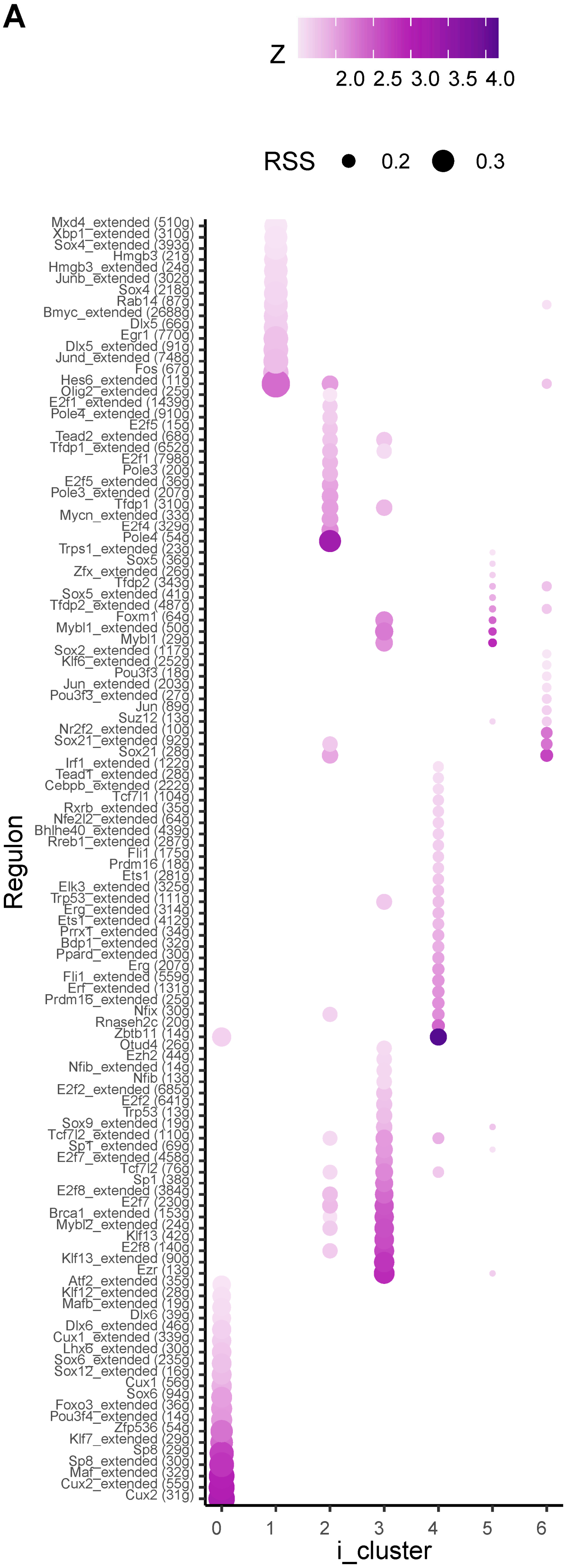
Z
2.0
2.5
3.0
3.5
4.0
RSS
0.2
0.3
Regulon
Mxd4_extended (510g)
Xbp1_extended (310g)
Sox4_extended (393g)
Hmgb3 (21g)
Hmgb3_extended (24g)
Junb_extended (302g)
Sox4 (218g)
Rab14 (87g)
Bmyc_extended (2688g)
Dlx5 (66g)
Egr1 (770g)
Dlx5_extended (91g)
Jund_extended (748g)
Fos (67g)
Hes6_extended (11g)
Olig2_extended (25g)
E2f1_extended (1439g)
Pole4_extended (910g)
E2f5 (15g)
Tead2_extended (68g)
Tfdp1_extended (652g)
E2f1 (798g)
Pole3 (20g)
E2f5_extended (36g)
Pole3_extended (207g)
Tfdp1 (310g)
Mycn_extended (33g)
E2f4 (329g)
Pole4 (54g)
Trps1_extended (23g)
Sox5 (36g)
Zfx_extended (26g)
Tfdp2 (343g)
Sox5_extended (41g)
Tfdp2_extended (487g)
Foxm1 (64g)
Mybl1_extended (50g)
Mybl1 (29g)
Sox2_extended (117g)
Klf6_extended (252g)
Pou3f3 (18g)
Jun_extended (203g)
Pou3f3_extended (27g)
Jun (89g)
Suz12 (13g)
Nr2f2_extended (10g)
Sox21_extended (92g)
Sox21 (28g)
Irf1_extended (122g)
Tead1_extended (28g)
Cebpb_extended (222g)
Tcf7l1 (104g)
Rxrb_extended (35g)
Nfe2l2_extended (64g)
Bhlhe40_extended (439g)
Rreb1_extended (287g)
Fli1 (175g)
Prdm16 (18g)
Ets1 (281g)
Elk3_extended (325g)
Trp53_extended (111g)
Erg_extended (314g)
Ets1_extended (412g)
Prrx1_extended (34g)
Bdp1_extended (32g)
Ppard_extended (30g)
Erg (207g)
Fli1_extended (559g)
Erf_extended (131g)
Prdm16_extended (25g)
Nfix (30g)
Rnaseh2c (20g)
Zbtb11 (14g)
Otud4 (26g)
Ezh2 (44g)
Nfib_extended (14g)
Nfib (13g)
E2f2_extended (685g)
E2f2 (641g)
Trp53 (13g)
Sox9_extended (19g)
Tcf7l2_extended (110g)
Sp1_extended (69g)
E2f7_extended (458g)
Tcf7l2 (76g)
Sp1 (38g)
E2f8_extended (384g)
E2f7 (230g)
Brca1_extended (153g)
Mybl2_extended (24g)
Klf13 (42g)
E2f8 (140g)
Klf13_extended (90g)
Ezr (13g)
Atf2_extended (35g)
Klf12_extended (28g)
Mafb_extended (19g)
Dlx6 (39g)
Dlx6_extended (46g)
Cux1_extended (339g)
Lhx6_extended (30g)
Sox6_extended (235g)
Sox12_extended (16g)
Cux1 (56g)
Sox6 (94g)
Foxo3_extended (36g)
Pou3f4_extended (14g)
Zfp536 (54g)
Klf7_extended (29g)
Sp8 (29g)
Sp8_extended (30g)
Maf_extended (32g)
Cux2_extended (55g)
Cux2 (31g)
0
1
2
3
4
5
6
i_cluster

Figure S5. Mouse active regulons within the integrated MGE clusters. We inferred the gene regulatory network based on co-expression and TF binding site enrichment separately for mouse MGE using SCENIC. The regulon specific scores (RSSs) predict levels of cell types specific to regulons in a given integrated MGE cell cluster.
